# Mineral Content, Functional, Thermo-Pasting, and Microstructural Properties of Spontaneously Fermented Finger Millet Flours

**DOI:** 10.3390/foods11162474

**Published:** 2022-08-17

**Authors:** Masala Mudau, Shonisani Eugenia Ramashia, Mpho Edward Mashau

**Affiliations:** Department of Food Science and Technology, Faculty of Science, Engineering and Agriculture University of Venda, Thohoyandou 0950, South Africa

**Keywords:** millets, techno-functional properties, gluten-free, natural fermentation, microstructure

## Abstract

Finger millet is a cereal grain which is superior to wheat and rice in terms of dietary fibre, minerals, and micronutrients. Fermentation is one of the oldest methods of food processing, and it has been used to ferment cereal grains such as finger millet (FM) for centuries. The aim of this study was to investigate the impact of spontaneous fermentation (SF) on mineral content, functional, thermo-pasting, and microstructural properties of light- and dark-brown FM flours. Spontaneous fermentation exhibited a significant increase in the macro-minerals and micro-minerals of FM flours. In terms of functional properties, SF decreased the packed bulk density and swelling capacity, and it increased the water/oil absorption capacity of both FM flours. Spontaneous fermentation had no effect on the cold paste viscosity of FM flours. However, significant decreases from 421.61 to 265.33 cP and 320.67 to 253.67 cP were observed in the cooked paste viscosity of light- and dark-brown FM flours, respectively. Moreover, SF induced alterations in the thermal properties of FM flours as increments in gelatinisation temperatures and gelatinisation enthalpy were observed. The results of pasting properties exhibited the low peak viscosities (1709.67 and 2695.67 cP), through viscosities (1349.67 and 2480.33 cP), and final viscosities (1616.33 and 2754.67 cP), along with high breakdown viscosities (360.00 and 215.33 cP) and setback viscosity (349.33 and 274.33 cP), of spontaneously fermented FM flours. Scanning electron microscopy showed that SF influenced changes in the microstructural properties of FM flours. The changes induced by SF in FM flours suggest that flours can be used in the food industry to produce weaning foods, jelly foods, and gluten-free products that are rich in minerals.

## 1. Introduction

Finger millet (*Eleusine coracana*) is a nutrient-dense cereal grain that is underutilised and is commonly viewed as food for poor people [1]. Globally, finger millet (FM) is important, ranking fourth after sorghum, pearl millet, and foxtail millet [2]. It is commonly cultivated in subtropical and tropical areas where it is difficult to grow other cereal crops such as maize and wheat [3]. In Africa, FM is widely cultivated in Kenya, Uganda, Ethiopia, Burundi, Malawi, Tanzania, and Zambia [4]. There are different cultivars of FM such as white, brown, and reddish cultivars [5]. The brown cultivar is used to produce traditional beer and porridge, while the white cultivar is utilised in the baking industry [6].

The grain contains no gluten and can be a good alternative for celiac patients [7]. It is high in micronutrients such as vitamins and minerals, as well as essential amino acids (tryptophan, methionine, histidine, and lysine) [8]. The layers of bran of FM consist of phenolic compounds which provide various nutritional and functional benefits [9]. Finger millet has nutraceutical characteristics, and it also has antimicrobial, antidiarrhea, antidiabetic, antioxidant, antitumour, and anti-inflammatory properties [10]. The nutritional content of FM is dependent on the processing methods and the presence or absence of antinutritional factors, including the interaction of nutrients with other food components. Antinutritional factors including oxalates, tannins, and phytic acid present in cereal grains such as FM have been reported to limit the nutrients’ bioavailability [11,12,13]. Cereal grains are subjected to different food processing techniques in the preparation or processing stages to reduce antinutritional factors.

Fermentation, malting, decortication, soaking, and germination are just a few of the old traditional food processing technologies that decrease antinutritional factors in cereal grains [14,15,16]. Spontaneous fermentation (SF) is a metabolic process that uses microorganisms to break down complex materials into simpler forms [17]. It is an ancient food processing technique that has been used for a long time, which enhances the functional, thermal, and pasting properties, as well as nutritional quality of foods [18,19,20]. Moreover, SF conspicuously improves the nutritional composition of cereal and millet grains since it enhances protein content, digestibility, and lysine content [21].

During the SF process, flour goes through various chemical changes such as modification of sugar, softening, and starch hydrolysis. This results in an improvement of the nutritional composition of FM grain, low levels of antinutritional factors, and the enhancement of micronutrients’ bioavailability [22]. Additionally, Azeez et al. [8] and Mutshinyani et al. [23] reported an increase in titratable acidity, total soluble solids, polyphenols (total phenolic and flavonoid contents), and antioxidant activity (DPPH and FRAP), along with a decrease in the pH value, of spontaneously fermented brown FM flours. The influence of SF on phenolic compounds depends on the types of grain, microorganism species, and fermentation conditions such as temperature, pH, and time. Low pH during SF extends the shelf-life of flours since the growth of most bacteria is inhibited at pH level less than 4 [9]. The decrease in pH of spontaneously fermented flours is due to soluble organic acids released from flours, which are products of lactic acid fermentation [23]. This shows that SF does impart desirable changes in the biochemical properties of food.

The functional properties of foods are important chemical and physical properties that show how structures, compositions, molecular conformation, and physicochemical properties interact with the environment, as well as the states in which they are determined and interrelated [24]. The behaviours of ingredients used for food preparation or cooking, as well as how they impact the taste and appearance of the product, are described by functional properties [25]. The functional properties of flour which include bulk density, oil, and water absorption capacity are essential determinants of bakery products’ quality [26]. They affect the sensory and textural properties, as well as the shelf-life, of food products [27]. Pasting properties determine the suitability of flours for baking applications [28].

There are limited studies on how SF affects the functional and thermo-pasting properties of FM flours. Modifications in the functional and microstructural properties of pearl millet flour due to SF have been reported [29,30,31]. Amadou et al. [18] reported changes in the pasting and thermal properties of foxtail millet caused by SF. It is, thus, critical to try to understand the impact of SF on the different components of both varieties of FM grains, which may contribute to the body of knowledge on the grain’s functionality. Such information may increase the utilisation of FM in the food industry and aid in the attainment of food security in third-world countries. Despite its superiority, FM is still underutilised in processed food products due to low usage in ready-to-eat or use food. Hence, the main aim of this paper was to investigate the influence of SF on the mineral content, as well as the functional, thermo-pasting, and microstructural properties, of FM flours.

## 2. Materials and Methods

### 2.1. Materials and Reagents

About 10 kg of each of light- and dark-brown FM grains were purchased from different street vendors at the local market of Thohoyandou, Limpopo Province, South Africa. Analytical-grade chemicals used for analysis were obtained from Merk Chemicals (PTY) Ltd., Germiston, South Africa.

### 2.2. Finger Millet Flour Production

All foreign matters (soil and stones) on FM grains were washed away with tap water, and the wet grains were placed on a tray wrapped with foil, transferred to an air oven drier, and allowed to dry for 24 h at the temperature of 40 °C. Some of the dry grains were reduced to flour with a miller (ZM 200 Miller, Retsch, Düsseldorf, Germany) and sifted with a 500 μm mesh sieve to make native flour, which was used as the control. The control sample was placed in an airtight plastic and kept in the refrigerator at 4 °C until it was needed for analysis [32]. For SF, the dried grains (200 g) were dispensed in a container filled with 800 mL of distilled water and spontaneously fermented in an incubator for different times (24, 48, and 72 h), at the temperature of 28 °C. The water was thrown away after each fermentation phase before the grains were transferred into an air oven drier for 24 h drying (40 °C). Dried grains were then pulverised with a miller (ZM 200 Miller, Retsch, Düsseldorf, Germany) and sifted through 500 μm mesh sieve to produce fermented flours. The fermented flours were transferred into a polyethylene and kept at 4 °C in refrigerator for further analysis [33]. For validation of the results, flour samples were replicated three times.

### 2.3. Mineral Compositions of Fermented Finger Millet Flours

ICP emission spectroscopy (ICP-AES, Jarrel-Ash) was employed to quantify the macro-elements and trace elements of FM flours as per method used by Ramashia et al. [34]. Approximately 2 g of flour samples were burned by a muffle furnace at 550 °C for 3 h until they were reduced to ashes. Thereafter, the ashes obtained were combined with 5 mL of nitric acid and 10 mL of hydrochloric acid solution. The combination was placed in the water bath for 1 h, mixed with 10 mL of HCL, and poured into a 100 mL of volumetric flask. After that, there was an addition of distilled water until a volume of 100 mL in the volumetric flask was reached. The sample was placed in ICP-AES for mineral analysis. Minerals were measured in milligrams per 100 g.

### 2.4. Functional Properties of Spontaneously Fermented Finger Millet Flours

#### 2.4.1. Loose/Packed Bulk Density

The loose/packed bulk density (LBD/PBD) of FM flours was measured as per the method proposed by Amandikwa et al. [35]. About 10 g of FM flours were weighed in a weighing boat and transferred to a 25 mL measuring cylinder to obtain LBD. The PBD was obtained by severally tapping the bottom of the cylinder until a constant volume of FM flours was observed. The flour weight per flour volume was used to calculate loose BD and packed BD (g/cm^3^).

#### 2.4.2. Water/Oil Absorption Capacity

The water absorption capacity (WAC) and the oil absorption capacity (OAC) of FM flours were measured as per the method described by Mudau et al. [36]. About 1 g of flour sample was weighed in a weighing boat, transferred into a weighed centrifuge tube, and combined with 10 mL of water/sunflower oil. The combination was vortexed (Model 36110740, Separation Scientific, Midrad, South Africa) for 30 min at room temperature (±25 °C) and centrifuged (Rotina 380 R- Labotech Ecotherm, Midrand, South Africa) at 3000× *g* rpm for 25 min. After centrifugation, the liquid above the sediment was poured into a beaker, and the WAC and OAC were calculated by subtracting the weight of the sample before and after the addition of water/sunflower oil from the weight of the sample. The obtained results were expressed in grams of water/sunflower oil per gram of FM flour.

#### 2.4.3. Swelling Capacity

The swelling capacity (SC) of FM flours was evaluated as described by Adebiyi et al. [29]. The flour was transferred into a 100 mL graduated cylinder until the 10 mL mark in the cylinder was reached. After that, distilled water was added until the 50 mL mark of total volume was reached. The tops of the graduated cylinders were then covered tightly and inverted so that the contents could be thoroughly mixed. After 2 min, the suspension was inverted again and allowed to settle for 30 min. After 30 min, the sample’s volume was taken.

#### 2.4.4. Viscosity of Finger Millet Flours

The viscosity of FM flours was examined using a Brookfield viscometer (Model RV, Brookfield Engineering, Inc.,Middleboro, MA, USA) according to a method described by Ramashia et al. [6]. About 10 g of FM flours were hydrated with distilled water (90 mL) in a beaker for 30 min. The mixture was agitated occasionally until it became a slurry of which the viscosity was measured, and the cold paste’s viscosity was recorded. The viscosity of cooked paste was determined by heating the slurry until it boiled at 95 °C in a water bath. The cooked paste was cooled to 30 °C and measured, and the viscosity was recorded.

### 2.5. Thermal Properties of Finger Millet Flours

The thermal properties of fermented FM flours were measured through differential scanning calorimetry (DSC) (DSC 4000, Perkin-Elmer, Shelton, CT, USA). A DSC pan was placed on weighing balance, and 25 mg of FM flours were transferred into it. The pan was sealed and heated from 20 °C to 130 °C. The heating rate was 10 °C per minute. In all DSC runs, there was an empty sealed pan serving as control. The gelatinisation temperatures (onset, peak, and conclusion temperature), gelatinisation temperature range, and the gelatinisation enthalpy of FM flours were evaluated and recorded through Pyris thermal system software connected to DSC [37].

### 2.6. Pasting Properties of Fermented Finger Millet Flours

A rapid Visco-analyser (RVA-4, Narrabeen, Australia) was employed to analyse the pasting properties parameters of FM flours as per the method explained by Siwatch et al. [38]. Briefly, 2.5 g of FM flour was dipped in 25 mL of distilled water inside the sample canister, which was then loaded into the RVA. The contents were heated to 50 °C for 1 min and heated to 90 °C again for 1 min before cooling for 2 min at 50 °C. The heating and cooling rates were constant at 12 °C per min. All pasting viscosities or parameters were recorded by a thermocline version 3.

### 2.7. Colour Profile of Spontaneously Fermented Finger Millet Flours

The colour of FM flours was analysed using Hunter Lab colorimeter (MiniScan XE Plus, Model CM-3500d, Hunter Associate laboratory, Reston, VA, USA) with a D65 light source, 8° observer, diffuse/O mode, 8 mm aperture of the instrument for illumination, and 8 mm for measurement. The equipment was calibrated with black and white tiles. The colour reading was expressed by Hunter values for L* (lightness), a* (redness), and b* (yellowness). The following formulas were used to calculate chroma (*C*), hue angle (*H*°), and colour change (Δ*E*):

$$Chroma=\sqrt{{\left(a*\right)}^{2}+{\left(b*\right)}^{2}},$$

$$Hue\;\left(H{}^{\circ}\right)=\tan^{-1}\{\frac{b*}{a*}\},$$

$$\Delta E=\sqrt{{\left(L-Lc\right)}^{2}+{\left(a-ac\right)}^{2}+{\left(b-bc\right)}^{2}},$$

where *Lc* is the lightness of the control sample, *ac* is the redness of the control sample, and *bc* is the yellowness of the control sample.

### 2.8. Fourier-Transform Infrared Spectra of Fermented Finger Millet Flours

An FTIR spectrometer Nicolet 8700 (Thermo Scientific, Inc., Santa Clara, CA, USA) was used to analyse FM flours, with wavelengths ranging from 400 to 4000 cm^−1^. About 0.5 g of flours were mounted on the instrument and analysed, and the sample’s spectra were obtained. The equipment ran 32 scans for each spectrum collected [29].

### 2.9. Microstructural Analysis of Fermented Finger Millet Flours Using Scanning Electron Microscopy (SEM)

A gold palladium layer in a coater was used to coat FM flour samples that were placed on a sample holder. A scanning electron microscopy (Model, JSM 6610-LV, Chicago, IL, USA) was used to analyse the microstructure of FM flours at 1000× magnification and 20 µm scale following a slightly modified method used by Gull et al. [39].

### 2.10. Statistical Analysis

The statistical tool SPSS 26 for windows (SPSS Inc., Chicago, IL, USA) was employed to assess the experimental data. The experimental data gathered from each of the FM flour samples was measured in triplicate. The disparities between the values of means were compared through Duncan’s multiple range test (*p* ≤ 0.05).

## 3. Results and Discussion

### 3.1. Mineral Composition of Spontaneously Fermented FM Flours

The impact of SF on the mineral composition of FM flours is shown in Table 1. The macro-minerals and some micro-minerals in the two flours increased as SF time increased. When compared to the native flours, the macro-minerals in the two flours were considerably higher at 72 h of fermentation. In light-brown FM flours, the sodium (Na) content significantly increased by 86.24%, followed by magnesium (Mg), phosphorus (*p*), potassium (K), and calcium (Ca), which increased by 14.26%, 7.05%, 6.70%, and 5.33%, respectively. The Ca, P, K, Mg, and Na values were also higher in 72 h dark-brown FM flours as compared to the native flours, with increases of 7.42%, 10.97%, 11.25%, 18.44%, and 161.87%, respectively. These results are consistent with those of Azeez et al. [8], who observed an increase in Ca and K in germinated and solid-state fermented FM flours.

For micro-minerals, there was a significant decrease in manganese (Mn) by 4.11% in 72 h light-brown FM flour in comparison with the native flour. Increases by 83.33%, 82.40%, and 76.86% were observed in the iron (Fe), copper (Cu), and zinc (Zn) of light-brown FM flours. After the 72 h fermentation period, the Zn and Mn contents in dark-brown FM flours were 12.10% and 19.43% lower than the native dark-brown FM flour, respectively. However, Cu and Fe were 165.90% and 57.40% significantly higher than the native flours. The increase in macro-mineral and some micro-mineral contents of fermented FM flours could be attributed to the decrease in antinutritional factors such as phytic acids that might have occurred during SF [8]. Mineral bioavailability can be reduced by antinutritional factors such as phytic acids, which form insoluble compounds with minerals. Hence, during SF, the enzymatic activities break down insoluble complexes between phytic acids and minerals, resulting in increased mineral content [24]. An improved mineral content due to SF has previously been reported [8,40,41]. Therefore, the enhanced mineral contents of light-brown and dark=brown FM flours by SF imply that both flours may be employed in the food industry to produce food products that can address mineral deficiencies in children, especially in most developing countries such as South Africa. Both flours can also be used to produce mineral-rich gluten free products such as biscuits.

### 3.2. Functional Properties of Fermented Finger Millet Flours

Table 2 presents the functional properties of spontaneously fermented FM flours. The loose bulk density of both FM flours was within the range of 0.47 to 0.56 g/mL. Statistically, SF had no impact on the loose bulk density of FM flours. The value of 0.47 g/mL is similar to that found by Elkhalifa et al. [42] for fermented sorghum flour. For PBD, a slight decrease with increasing period of SF was observed in both FM flours. The declining trend could be due to the SF of FM grains, which often results in the breakdown of proteins and carbohydrates into small units [29]. The lower bulk density of fermented FM flour can help with the formulation of reduced bulk weaning foods for babies [43].

The lower bulk density of fermented FM flour can help with the formulation of reduced bulk weaning foods for babies [43].

The WAC is a measurement of how flour and water interact in a variety of foods [44]. The presence of hydrophilic groups which bind water in flour determines the ability of that flour to absorb water [34]. The WAC values in 72 h FM flours were significantly higher (*p* < 0.05) than those in other samples. This could be due to the higher presence of polar amino acids and hydrophilicity of carbohydrates [45], as well as the loss of starch polymers caused by SF, resulting in 72 h FM flours possessing high WAC [29]. Igbabul et al. [32] argued that high WAC is good for making bakery products. Furthermore, the low WAC obtained in native FM flours suggests a lower presence of carbohydrates and low water-binding hydrophilic groups. Adebowale et al. [46] stated that a lower WAC value suggests starch polymer structural compactness. Tenagashaw et al. [47] noted that a lower WAC of flour is good for producing thin gruels for baby formulas. Awuchi et al. [25] found that the flour’s WAC is important for baking, product homogeneity, and bulking.

The OAC of both FM flours increased as SF time increased, and the highest OAC was obtained in 72 h fermented flours, while the lowest OAC was obtained in native FM flours. The increasing trend of OAC observed suggest that fermented FM flours may contain more hydrophobic proteins with better lipid-binding properties; hence, an increase in OAC was observed in fermented FM flours. Protein is the most important chemical component affecting OAC, and the chains of amino acids (alanine, leucine, etc.) that are nonpolar interact with chains of hydrocarbon lipid [48]. The OAC increment of fermented flours observed was consistent with the study of Adebiyi et al. [29], who noted an increment of OAC in pearl millet flour due to fermentation. A higher OAC observed means that fermented FM flours have the potential to be used in food preparation because such a quality retains flavour and improves mouth feel [49].

Ayo-Omogie and Ogunsakin [50] argued that the SC of flour is a crucial parameter influencing the consistency of the flour, and it is impacted by the compositional structure of the flour. The SC of both FM flours ranged between 13.33 and 14.33 mL. A higher SC was obtained in native (0 h) FM flour, while a lower SC was obtained in 72 h fermented FM flours. The SC of 72 h FM flour was reduced by SF, and these findings were similar to those obtained by Adebowale and Maliki [51]. In their study, the SC ofdecreased after SF. Olukomaiya et al. [52] reported that a higher SC indicates an enhancement of flour functionality, which would eventually result in a good food product. Ayo-Omogie and Ogunsakin [50] mentioned that a lower SC suggests that fermented FM flour can produce a nutritionally dense food for infants.

### 3.3. Viscosity of Spontaneously Fermented Finger Millet Flours

**Figure 1:**
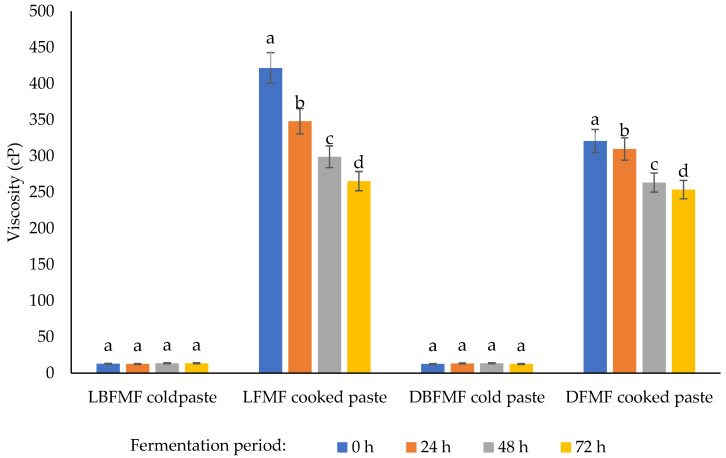
Influence of spontaneous fermentation on cold paste and cooked paste of finger millet flours. Different letters show statistically different effects (*p* < 0.05). LBFMF = light-brown finger millet flour; DBFMF = dark-brown finger millet flour.

The viscosity of spontaneously fermented FM flours is shown in Figure 1. According to the cold paste results, there were no significant differences (*p* < 0.05) observed among the flour samples. The cooked paste viscosity for native FM flour was significantly higher than that of spontaneously fermented FM flours. Uvere et al. [53] noted that the decreasing trend observed in fermented flours might be attributed to starch granule hydrolysis due to amylase activity during SF. Many studies have also found that the tendency of starch hydrolysis to form sugars results in the reduced viscosity of flour samples in various food processing technologies [11,32,43,54]. Uyere et al. [53] stated that a decrease in viscosity in fermented FM flours could also be due to granule structural degradation caused by milling, resulting in an increase in the amount of soluble materials such as dextrin with a short chain and some sugars.

Usman et al. [55] stated that the low viscosity of fermented FM flours indicates that the flour is suitable for making weaning foods for new-borns. Both cold and cooked paste values obtained in native FM flour were similar to those obtained by Ramashia [56] and Pawase et al. [57] for fortified and unfortified FM flours. These findings were also in consistent with Onweluzo and Nwabugwu [33], who studied how SF affects the viscosity of pearl millet flour.

### 3.4. Thermal Properties of Spontaneously Fermented Finger Millet Flours

Table 3 summarises the impact of SF on the thermal properties of FM flours. The gelatinisation temperatures (onset temperature (T_o_), peak temperature (T_p_), and conclusion temperature (T_c_)) of both FM flours increased with the increasing period of SF. Higher gelatinisation temperatures were observed in 72 h fermented flours. The increase in peak temperature after fermentation might be attributed to the accumulation of proteolytic enzymes generated by natural bacteria that break down the cell walls of grain, resulting in more starch released and higher crystallite structure proportions in the sample [38]. Kumoro and Hadyiyat [58] stated that the SF process can alter the macromolecular structure or arrangement of amylopectin and amylose in flour granules, changing gelatinisation temperatures.

A similar increase in onset temperature (T_o_) and peak temperature (T_p_) after fermentation was observed by Amadou et al. [16], Kumoro and Hadiyat [58], and Olamiti et al. [59] for foxtail millet, Korean wheat, and pearl millet flours. The inherent modifications of granule size, morphology, starch distribution, and internal starch fraction organisation inside the granules could explain the disparities in To, Tp, and Tc values identified in fermented FM flours [60].

High gelatinisation temperatures obtained in 72 h fermented FM flours suggested that more energy was required to start the starch gelatinisation process. The increase in gelatinisation temperatures of fermented flours could be due to the production of amino acids resulting from protein alteration during SF [8]. However, starches with low gelatinisation temperatures, such as those found in native and 24 h fermented flours are best known for excellent cooking quality [61].

In terms of gelatinisation temperature range (T_r_), there was no significant different observed among the samples. The gelatinisation enthalpy (∆H) of both FM flours decreased as SF time increased. This decrease means that fermented flours would require less energy to disrupt starch granules bonds [62]. A similar decreasing trend of ∆H was also observed by Ahmed et al. [63] and Bian et al. [64], for fermented koreeb seed flours and proso millet flour.

### 3.5. Pasting Properties of Spontaneously Fermented Finger Millet Flours

The pasting properties of FM flours are used to evaluate if the flour is suitable for use in baking [56]. They also reflect the variations in flour viscosity that occur when it is heated in excess water while being constantly stirred [64]. Table 4 summarises the effect of SF on pasting properties of FM flours. The peak viscosity (PV) decreased with the increasing period of SF and later started to increase at 72 h. The PV indicates the maximum SC of starch granules and WAC [31]. The higher PV observed in native FM flour was probably due to the flour having a higher content of starch [65]. The lower PV indicates that the fermented FM flours have lower thickening power. The shorter starch chains formed during SF could be responsible for the decrease in PV [66]. The PV values obtained in native flour were similar to those obtained by Dasa and Binh [67] for a variety of millet flour.

The trough viscosity (TV) obtained from native flour was higher than that obtained from fermented FM flours. The TV reflects the gel or viscous paste-forming capacity of the flour after heat treatment and how well it can withstand stress generated by stirring [68]. Therefore, the lower TV observed in fermented flours suggests the shear resistance of swollen granules.

The breakdown viscosity (BDV) value determines how easily swelling granules can be dissolved, and this indicates the flour product’s stability [69]. It is also the difference between peak viscosity and through viscosity. The BDV of light-brown FM flour increased with an increasing period of SF up to 48 h period and started to decrease at 72 h. In dark-brown FM flour, the BDV increased with an increasing period of SF. The increment in BDV of fermented flours as compared to the native flours could be linked to the increase in protein content due to SF [18,70]. Similarly, Geng et al. [71] found an increase in BDV after the fermentation of rice. A higher BDV viscosity indicates a poorer capacity of the sample to endure the heat and shear stress generated by cooking [72,73]. The lower BDV of both native FM flours indicates the resistance of the starch to thermal treatment and shearing.

The final viscosity (FV) values of native FM flours were higher than those of fermented FM flours. The decrease in FV observed in fermented flours could be ascribed to the breakdown of amylose into sugars during SF [74]. The lower FV values observed in fermented FM flour denotes a loss of the capacity of flours to make a viscous paste. According to Oloyede et al. [73], FV represents the starch’s capacity to form a gel and paste in the flour after heat treatment.

The setback viscosity (SV) is a retrogradation index that is linked to the amylose content [75]. A higher setback value indicates a greater likelihood of amylose retrograding and forming a gel structure [74]. The findings in this study showed that SF increased the SV values of FM flours, and this could be due to amylose’s ability to rearrange itself after being disrupted. A similar increase in SV values after fermentation was found by Said et al. [66] for rice flour. The high values of SV obtained in fermented flours imply that the flours could be good for making noodles and jelly foods [76].

Peak time determines cooking time [77]. The highest peak time in this study was found in 72 h fermented FM flours, while the lowest peak time was found in native flours. This result suggested that 72 h fermented FM flours require more cooking time than native flours. Oyeyinka et al. [78] observed a similar increase in peak time values as fermentation progressed. Other studies reported similar values of peak time [79,80].

### 3.6. Colour Attributes of Finger Millet Flours

The colour of light- and dark-brown FM flours was measured in terms of L*, a*, and b* values as shown in Table 5. The L* values of the two flours increased with an increasing period of fermentation. A higher L* value was obtained in 72 h fermented flours, while a lower L* value was obtained in native flours. The increase in the lightness of flour samples could be due to dissolution of coloured (red or yellow) pigments caused by changes in the carbohydrates or protein hydrolysate during fermentation [81]. 

Akinola et al. [82] also showed an increase in the lightness of pearl millet flour caused by fermentation. The results of this study were also similar to those obtained by Siroha et al. [83] and Ramashia [56] for different cultivars of FM flours.

Higher a* values were obtained in native light- and dark-brown FM flours while lower a* values were obtained in 72 h fermented FM flours. This means that both native light- and dark-brown FM flours contained higher red pigmentation. In both cultivars, however, there was no significance difference (*p* < 0.05) between 24 and 48 h fermented FM flours. The findings in this investigation are similar to those of Olamiti et al. [59] for malted and fermented pearl millet flours. The b* values were higher in both native FM flours and lower in 72 h fermented flours. Positive b* values obtained in this study showed the yellow pigmentation of both FM flours. Similar results of b* values were also obtained by Siroha et al. [83] for millet flours. According to a* and b* results, fermented flours were less red and yellow as compared to the native flours.

The chroma values were higher in native flours than in fermented flours. The hue angle (H°) values of the two flours increased significantly with increasing period of fermentation.

According to Mudau et al. [70], yellow, green, and blue hues are represented by angles of 90°, 180°, and 270°, respectively, whereas red hue is represented by a hue angle of 0° or 360°. Therefore, all the flour samples were closer to red. The colour difference (ΔE) values of 72 h fermented flours as compared to natives were significantly higher, indicating that fermentation changed the colour of flours.

### 3.7. Fourier-Transform Infrared Spectra of Spontaneously Fermented Finger Millet Flours

**Figure 2:**
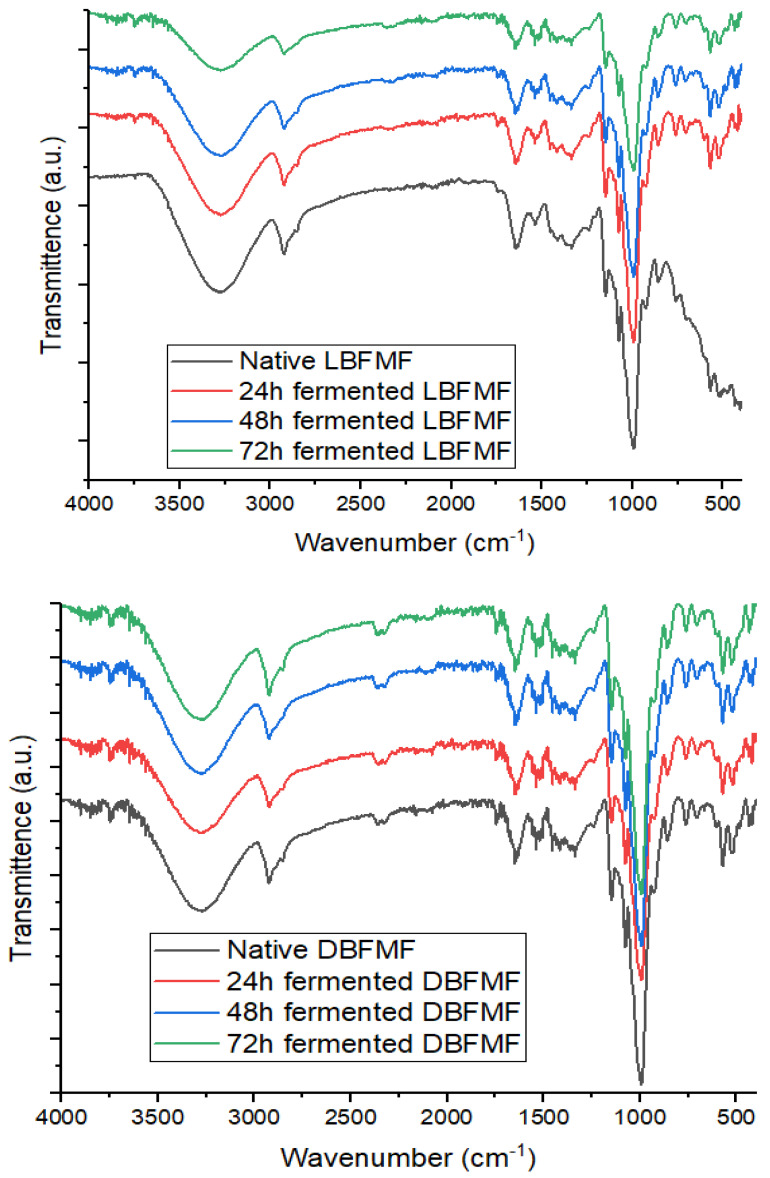
FTIR spectra of finger millet flours. LBFMF = light-brown fermented finger millet flours, DBFMF = dark-brown fermented finger millet flours.

Figure 2 shows the FTIR spectral regions (O–H stretch region, C–H stretch region, and fingerprint region) of light- and dark-brown FM flours. The O–H region of light-brown FM flours showed bands (peaks) within the range of 3273.45 to 3273.92 cm^−1^. In dark-brown FM flours, the absorption peaks were in the range of 3274.25 to 3272.74 cm^−1^. The observed peaks could be ascribed to the stretching vibrations of O–H, and differences in peaks could be linked to variations of moisture content of FM flours caused by fermentation, as reported by Mudau et al. [70]. Moisture content has been reported to be responsible for the peaks in O–H stretch region [28,83]. Higher peak values observed in fermented flours suggest the presence of more alcohol produced during fermentation.

The C–H band’s width increased in the two flours as the fermentation period increased, with absorption peaks ranging from 2924.29 to 2925.35 cm^−1^ and 2924.47 to 2925.49 cm^−1^, respectively. This was probably caused by the stretching vibrations of aromantic and aliphatic C–H bonds [30]. The variation in the peaks could be correlated to differences observed in the fat content of fermented FM flours as reported by Mudau et al. [70]. They were also peaks within the range of 1644.55 to 1651.86 cm^−1^, denoting the presence of amide I (stretching and bending vibrations of C=O).

The stronger amide I peak intensities in the fermented flours could be linked to the increment in protein content caused by the fermentation of FM flours, as observed by Mudau et al. [70]. Series of bands in the fingerprint region (1500–900 cm^−1^) were also observed in this study. The peaks at around 1338.26 in native and fermented flours showed that the flours contained O–C–H, C–C–H, and C–O–H [84]. Other notably range of peaks in the FM region were observed near 1149.40 to 1148.02 cm^–1^, 1076.40 to 1076.20 cm^−1^, and 994.33 to 993.10 cm^−1^, revealing that C–O, C=O, and C–H functional groups were present in FM flours, as similarly reported by Navyashree et al. [81]. The disparities in peaks between 1148.85 and 994.25 994.25 cm^−1^ could be attributable to the stretching and bending vibrations of C–H, C–O–C, and C–O bonds [85], as well as the differences in carbohydrate and protein contents caused by different periods of fermentation [69]. Other absorbance peaks ranging from 859.69 to 406.94 cm^−1^ denoted the presence of COH, CCH, and OCH in the flours [86], and the variations among the peaks could be linked to changes in the protein content of flours caused by fermentation [71]. Adebiyi et al. [30] also linked the disparities of peaks to protein changes due to fermentation.

### 3.8. Scanning Electron Microscopy of Spontaneously Fermented Finger Millet Flours

**Figure 3:**
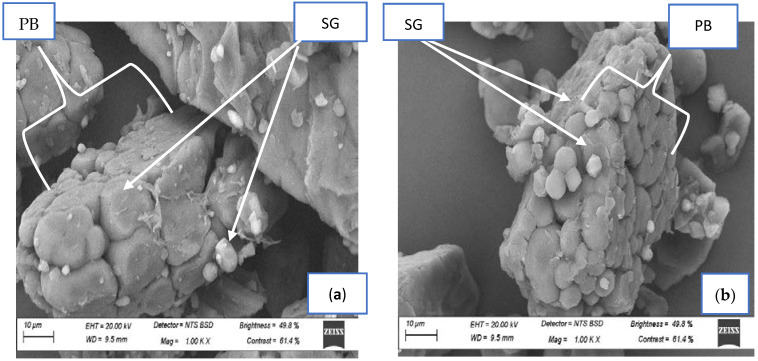

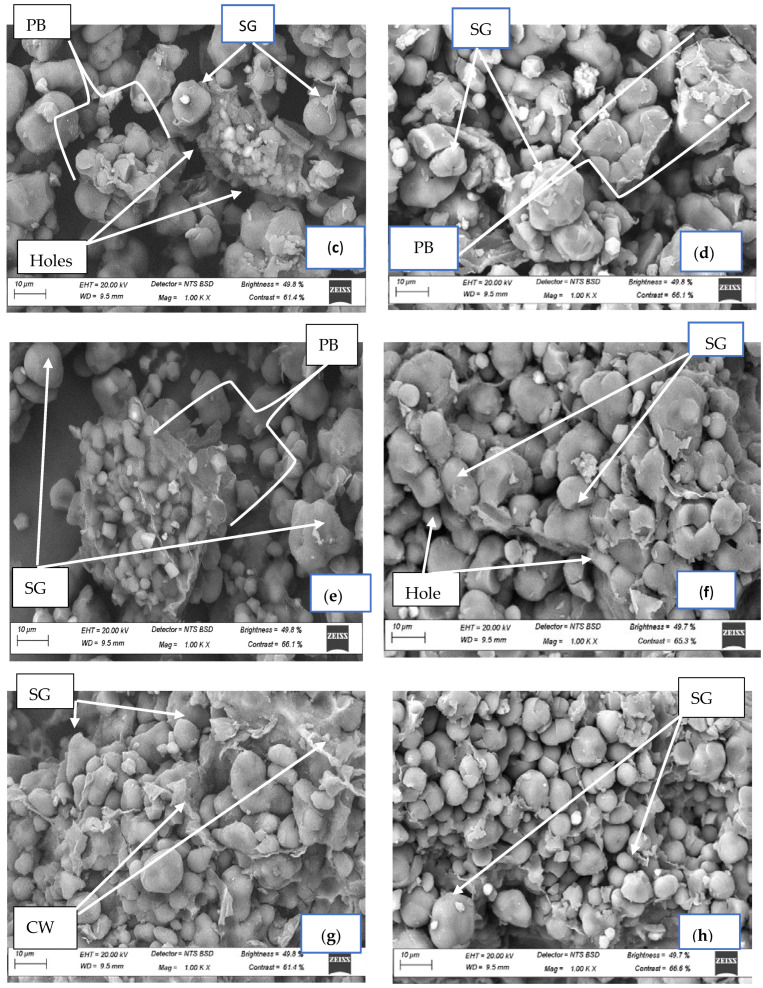
Scanning electron micrographs of fermented light- and dark-brown FMF: (**a**) native light-brown FMF; (**b**) native dark-brown FMF; (**c**) 24 h fermented light-brown finger millet; (**d**) 24 h fermented dark-brown FMF; (**e**) 48 h fermented light-brown FMF; (**f**) 48 h fermented dark-brown FMF; (**g**) 72 h fermented light-brown FMF; (**h**) 72 h fermented dark-brown FMF; PB = protein bodies; SG = starch granules; CW = cell walls; FMF = finger millet flours.

Figure 3 shows the scanning electron micrographs of fermented FM flours. The native light-brown FM flour showed a large compact structure of protein bodies (PBs), which was broken down during SF. The starch granules (SGs) in both native flours varied from small to large and were attached to each other in PBs. The subsequent fermented FM flours showed small PBs and more liberated SGs compared to the native flours that had a large structure of PBs with entrapped SGs. This could be due to the SF breaking down a large complex compound into simple molecules. The liberated SGs in the fermented flours varied from small to large with round and oval shapes.

There were also more holes observed between the SGs of fermented flours compared to the native flours. Narayanasamy [1] also found pits in fermented samples and attributed this to the enzymatic degradation of SG by microorganisms during SF. The total liberation of SGs was observed in both 72 h fermented FM flours, and this was probably due to the breaking of cell walls caused by SF. Salmenkallio-Marttila et al. [87] indicated that the breakdown of the cell wall has an impact on the microstructure of starch granules. Nainggolan et al. [88] observed similar results of SG for fermented cassava flour. Many studies have also linked microstructural alterations to fermentation [89,90,91]. Changes or loosening of the starch structure observed in FM flours due to SF could have been the reason for the observed disparities in the thermal properties, SC, WAC, and AOC of the flour. Khoza et al. [92] proposed a relationship between the morphology of flour and the SC, WAC, and thermal properties of the flour.

## 4. Conclusions

In this study, SF positively influenced the functional and thermo-pasting properties, as well as the mineral content, of both cultivars of FM flours. Spontaneous fermentation were shown to be an effective treatment in reducing packed bulk density, swelling capacity, cooked paste viscosity, peak viscosity, and trough viscosity of FM flours, which would make hem suitable to produce weaning foods or nutritionally dense foods. The improved water/oil absorption capacity, setback viscosity, and pasting temperature of fermented FM flours (particularly after 72 h) make the flours good for utilisation during manufacturing of bakery products, jelly foods, and thickening foods. The superiority of 72 h fermented flours in terms of minerals suggests that flours should be used to make foods that addresses mineral deficiencies in children, as well as celiac and elderly individuals. Fermentation also modified the microstructural properties of FM flours. Fermented and native FM flours are prospective raw materials to substitute wheat flour for gluten-free products with desirable properties. Therefore, future studies should focus on developing new gluten-free food products from fermented FM flours for people who suffer from celiac disease.

## Figures and Tables

**Table 1:** Impact of spontaneous fermentation on macro and micro-minerals of finger millet flours (mg/100 g dry basis).

	Fermentation Period (h)			
FM Cultivars	0	24	48	72
LBFMF				
Macro-minerals				
Ca	373.68 ± 1.67 ^a^	382.22 ± 1.34 ^b^	389.57 ± 1.95 ^c^	394.24 ± 1.48 ^d^
P	271.34 ± 1.61 ^a^	283.15 ± 1.61 ^b^	286.11 ± 0.53 ^c^	290.72 ± 1.17 ^d^
K	416.20 ± 1.91 ^a^	426.72 ± 1.36 ^b^	433.72 ± 0.73 ^c^	444.09 ± 1.58 ^d^
Mg	124.33 ± 1.27 ^a^	131.60 ± 1.89 ^b^	136.30 ± 0.89 ^c^	141.99 ± 2.16 ^d^
Na	9.88 ± 1.49 ^a^	14.47 ± 0.99 ^b^	16.83 ± 0.87 ^c^	18.43 ± 0.78 ^d^
Micro-minerals				
Cu	0.46 ± 0.12 ^a^	0.48 ± 0.26 ^b^	0.60± 0.07 ^b^	0.84 ± 0.09 ^c^
Zn	2.12 ± 0.12 ^a^	2.98 ± 0.27 ^b^	3.63 ± 0.10 ^c^	3.75 ± 0.22 ^d^
Fe	4.26 ± 1.07 ^a^	6.21 ± 1.33 ^b^	6.67 ± 0.54 ^b^	7.81 ± 0.29 ^c^
Mn	6.21 ± 0.52 ^a^	5.93 ± 1.12 ^a^	5.90 ± 0.74 ^a^	5.95 ± 0.54 ^a^
DBFMF				
Macro-minerals				
Ca	308.66 ± 3.06 ^a^	320.64 ± 2.35 ^b^	325.85 ± 1.70 ^c^	331.98 ± 1.36 ^d^
P	315.77 ± 0.52 ^a^	325.55 ± 0.77 ^b^	338.57 ± 0.70 ^c^	350.58 ± 0.87 ^d^
K	360.92 ± 0.84 ^a^	379.64 ± 0.44 ^b^	392.68 ± 0.74 ^c^	401.90 ± 1.55 ^d^
Mg	144.31 ± 1.27 ^a^	153.07 ± 0.17 ^b^	164.61 ± 0.82 ^c^	171.11 ± 1.89 ^d^
Na	5.66 ± 1.73 ^a^	10.94 ± 0.68 ^b^	13.36 ± 1.66 ^c^	14.83 ± 1.12 ^c^
Micro-minerals				
Cu	0.44 ± 0.11 ^a^	0.44 ± 0.05 ^a^	0.67 ± 0.10 ^b^	1.17 ± 0.08 ^c^
Zn	1.64 ± 0.05 ^b^	1.45 ± 0.18 ^a^	1.45 ± 0.28 ^a^	1.44 ± 0.11 ^a^
Fe	5.07 ± 0.35 ^a^	5.69 ± 0.59 ^a^	5.28 ± 1.01 ^a^	7.98 ± 0.33 ^d^
Mn	16.62 ± 1.15 ^b^	16.49 ± 0.32 ^b^	13.60 ± 0.70 ^a^	13.34 ± 1.02 ^a^

Values are expressed as the mean ± standard deviation. A row with different superscript letters indicates a significant difference (*p* < 0.05). P = phosphorus, Ca = calcium, Na = Sodium, K = potassium, Mg = magnesium, Mn = manganese, Fe = iron, Cu = copper, Zn = zinc. FM = finger millet, LBFMF = light-brown finger millet flour, DBFMF = dark-brown finger millet flour.

**Table 2:** Functional properties of spontaneously fermented finger millet flours.

Fermentation Period (h)	LBD (g/g)	PBD (g/g)	WAC (g/g)	OAC (g/g)	SC (mL)
LBFMF					
0	0.55 ± 0.07 ^a^	0.79 ± 0.04 ^a^	1.96 ± 0.10 ^a^	1.20 ± 0.28 ^a^	14.33 ± 0.58 ^a^
24	0.56 ± 0.00 ^a^	0.76 ± 0.01 ^ab^	2.05 ± 0.06 ^ab^	1.28 ± 0.09 ^b^	14.00 ± 1.00 ^a^
48	0.56 ± 0.03 ^a^	0.75 ± 0.02 ^ab^	2.07 ± 0.01 ^ab^	1.36 ± 0.15 ^b^	14.00 ± 1.00 ^a^
72	0.52 ± 0.02 ^a^	0.73 ± 0.03 ^b^	2.10 ± 0.03 ^b^	1.44 ± 0.17 ^c^	13.33 ± 0.58 ^b^
DBFMF					
0	0.47 ± 0.04 ^a^	0.77 ± 0.00 ^b^	2.05 ± 0.08 ^a^	1.24 ± 0.15 ^a^	14.00 ± 0.00 ^a^
24	0.50 ± 0.03 ^a^	0.74 ± 0.03 ^ab^	2.06 ± 0.01 ^a^	1.30 ± 0.10 ^b^	13.67 ± 0.58 ^a^
48	0.52 ± 0.02 ^a^	0.74 ± 0.02 ^ab^	2.08 ± 0.01 ^a^	1.35 ± 0.20 ^c^	13.67 ± 0.57 ^a^
72	0.49 ± 0.04 ^a^	0.71 ± 0.00 ^a^	2.14 ± 0.05 ^b^	1.39 ± 0.11 ^d^	13.33 ± 0.58 ^b^

Values are expressed as the mean ± standard deviation. A column with different letters indicates a significant difference (*p* < 0.05). LBD = loose bulk density, PBD = packed bulk density, WAC = water absorption capacity, OAC = oil absorption capacity, SC = swelling capacity, LBFMF = light-brown finger millet flour, DBFMF = dark-brown finger millet flour.

**Table 3:** Thermal properties of spontaneously fermented finger millet flours.

Fermentation Period (h)	T_o_ (°C)	Tp (°C)	Tc (°C)	Tr (°C)	∆H (J/g)
LBFMF					
0	76.64 ± 1.44 ^a^	78.41 ± 1.16 ^a^	79.99 ± 1.50 ^a^	3.35 ± 1.60 ^a^	5.07 ± 0.79 ^ab^
24	79.24 ± 0.81 ^b^	80.97 ± 0.82 ^b^	82.25 ± 1.48 ^b^	3.01 ± 0.67 ^a^	4.30 ± 0.54 ^b^
48	81.12 ± 1.25 ^bc^	82.06 ± 0.97 ^c^	85.01 ± 1.29 ^c^	3.88 ± 1.22 ^a^	4.28 ± 1.20 ^ab^
72	82.47± 0.89 ^c^	84.48 ± 4.58 ^d^	86.39 ± 0.56 ^d^	3.92 ± 1.43 ^a^	2.99 ± 0.22 ^a^
DBFMF					
0	69.42 ± 1.00 ^a^	70.98 ± 0.93 ^a^	78.24 ± 2.95 ^a^	8.82 ± 1.96 ^a^	4.87 ± 1.92 ^c^
24	70.10 ± 0.25 ^b^	72.21 ± 1.04 ^ab^	78.89 ± 1.67 ^a^	8.79 ± 1.43 ^a^	4.50 ± 0.84 ^b^
48	71.03 ± 0.60 ^c^	73.62 ± 1.87 ^bc^	79.95 ± 1.20 ^b^	8.92 ± 0.66 ^a^	4.38 ± 1.06 ^ab^
72	72.04 ± 1.05 ^d^	75.67 ± 0.68 ^c^	81.63 ± 0.80 ^c^	9.59 ± 0.61 ^a^	3.85 ± 0.85 ^a^

Values are expressed as the mean ± standard deviation. Different superscript letters in the column indicate a significant difference (*p* < 0.05). T_o_ = onset temperature, T_p_ = peak temperature, T_c_ = conclusion temperature, T_r_ = gelatinisation temperature range, ∆H = gelatinisation enthalpy, LBFMF = light-brown finger millet flour, DBFMF = dark-brown finger millet flour.

**Table 4:** Pasting properties of spontaneously fermented finger millet flours.

Fermentation Period (h)	Peak Viscosity (cP)	Trough Viscosity (cP)	Breakdown Viscosity (cP)	Final Viscosity (cP)	Setback Viscosity (cP)	Peak Time	PT (°C)
LBFMF							
0	2410.67 ± 48.23 ^a^	2308.67 ± 53.59 ^a^	102.00 ± 7.94 ^c^	2414.00 ± 66.84 ^a^	105.33 ± 18.01^d^	5.11 ± 0.75 ^c^	74.82 ± 0.45 ^a^
24	2111.67 ± 24.01 ^c^	1974.33 ± 35.59 ^b^	137.33 ± 12.34 ^b^	2181.00 ± 15.72 ^b^	206.67 ± 20.60 ^c^	5.24 ± 0.10 ^c^	75.05 ± 0.05 ^b^
48	1709.67 ± 7.23^d^	1349.67 ± 12.05^d^	360.00 ± 18.73 ^a^	1616.33 ± 7.51 ^c^	266.67 ± 7.37 ^b^	6.00 ± 0.13 ^b^	75.13 ± 0.32 ^b^
72	2246.67 ± 12.58 ^b^	1889.67 ± 4.73 ^c^	357.00 ± 17.35 ^a^	2239.00 ± 11.36 ^b^	349.33 ± 16.07 ^a^	6.69 ± 0.08 ^a^	75.25 ± 0.43 ^b^
DBFMF							
0	2876.67 ± 30.99 ^a^	2739.67 ± 32.01 ^a^	137.00 ± 2.73 ^d^	2959.00 ± 34.66 ^a^	219.33 ± 4.26 ^b^	6.07 ± 0.07 ^c^	75.02 ± 0.02 ^a^
24	2776.00 ± 25.51 ^b^	2652.33 ± 33.08 ^b^	123.67 ± 14.19 ^c^	2871.33 ± 32.96 ^b^	219.00 ± 7.81 ^b^	6.13 ± 0.07 ^c^	75.28 ± 0.23 ^b^
48	2762.00 ± 15.72 ^b^	2578.67 ± 18.58 ^c^	183.33 ± 33.62 ^b^	2874.67 ± 27.15 ^b^	296.00 ± 45.43 ^a^	6.40 ± 0.00 ^b^	75.42 ± 0.52 ^c^
72	2695.67 ± 11.59 ^c^	2480.33 ± 10.69 ^d^	215.33 ± 2.88 ^a^	2754.67 ± 10.97 ^c^	274.33 ± 7.57 ^a^	6.80 ± 0.07 ^a^	75.73 ± 0.43 ^d^

Values are expressed as the mean ± standard deviation. Different letters in the column indicate a significant difference (*p* < 0.05). LBFMF = light-brown finger millet flour, DBFMF = dark-brown finger millet flour.

**Table 5:** Effect of fermentation on the colour properties of spontaneously fermented finger millet flours.

Fermentation Period (h)	L*	a*	b*	Chroma	Hue Angle	ΔE
Light-brown FM flours						
0	75.05 ± 0.21 ^a^	4.28 ± 0.21 ^c^	7.32 ± 0.05 ^d^	8.48 ± 0.03 ^b^	59.67 ± 0.25 ^a^	0.00 ± 0.00 ^a^
24	78.04 ± 0.34 ^b^	3.32 ± 0.34 ^b^	6.72 ± 0.09 ^a^	7.50 ± 0.09 ^a^	63.70 ± 0.38 ^b^	3.19 ± 0.35 ^b^
48	78.75 ± 0.27 ^c^	3.26 ± 0.27 ^b^	6.85 ± 0.07 ^b^	7.58 ± 0.06 ^a^	64.58 ± 0.38 ^c^	3.86 ± 0.28 ^c^
72	83.24 ± 0.09 ^d^	2.62 ± 0.09 ^a^	7.00 ± 0.03 ^c^	7.48 ± 0.07 ^a^	69.46 ± 0.03 ^d^	8.35 ± 0.08 ^d^
Dark-brown FM flours						
0	72.07 ± 0.25 ^a^	3.54 ± 0.03 ^c^	8.36 ± 0.04 ^d^	9.07 ± 0.02 ^d^	67.03 ± 0.26 ^a^	0.00 ± 0.00 ^a^
24	74.56 ± 0.26 ^b^	2.99 ± 0.06 ^b^	7.95 ± 0.07 ^c^	8.49 ± 0.08 ^c^	69.36 ± 0.33 ^b^	2.59 ± 0.27 ^b^
48	74.83 ± 0.35 ^b^	3.04 ± 0.01 ^b^	8.10 ± 0.05 ^b^	8.65 ± 0.04 ^b^	69.48 ± 0.14 ^b^	2.82 ± 0.34 ^b^
72	76.08 ± 0.42 ^c^	2.79 ± 0.04 ^a^	7.82 ± 0.06 ^a^	8.30 ± 0.06 ^a^	70.34 ± 0.41 ^c^	4.11 ± 0.40 ^c^

Mean ± standard deviation. Different letters in the columns indicate a significant difference (*p* < 0.05) in mean values. L* = lightness, a* = redness, b* = yellowness, FM = finger millet.

## Data Availability

The data are available from the corresponding author.
